# Supplementary material for: Examining inequalities in access to delivery by caesarean section in Nigeria
Source: PLoS One. 2019 Aug 29;14(8):e0221778. doi: 10.1371/journal.pone.0221778 (PMC6715280; doi:10.1371/journal.pone.0221778)
Supplement: Attachment — Submitted filename: Dear Editor.docx [file pone.0221778.s001.docx]

Dear Editor,

Thank you for swiftly reviewing our manuscript.

**Comment**

I think this is a very relevant topic, inequity of in the availability of CS and in a country that makes a significant contribution to global maternal mortality. Given that the 2018 Nigeria DHS report and data sets are available, it makes your paper based on data related to 5 years preceding 2013 NDHS less relevant. What about running a similar analysis using DHS 2018 data set and comparing both results? If you can do this, I will be happy to send your revised manuscript for review.

Response

The comments are important and we made effort to use the latest nationally representative data available. We have now contacted DHS and were informed that the NDHS 2018 data is not available to the public at the moment and were also told that only the preliminary result is publicly available. The full report is not yet out. Given the paucity of studies on this important topic, we believe that our paper will contribute to the literature and serve as reference point to future studies. We have however noted the comments and added it as one of our future papers to be written once the NDHS 2018 dataset is finally released.
